# Efficacy of a smartphone-based intervention — “Holidaily” — promoting recovery behaviour in workers after a vacation: study protocol for a randomised controlled trial

**DOI:** 10.1186/s12889-020-09354-5

**Published:** 2020-08-26

**Authors:** Alexandra Smyth, Jessica de Bloom, Christine Syrek, Markus Domin, Monique Janneck, Jo Annika Reins, Dirk Lehr

**Affiliations:** 1 Department of Health Psychology and Applied Biological Psychology, Institute of Psychology, Leuphana University of Lueneburg, Universitaetsallee 1, 21335 Lueneburg, Germany; 2 Department of Human Resource Management and Organizational Behavior, University of Groningen, Groningen, Netherlands; 3 Department of Psychology, Tampere University, Tampere, Finland; 4 Department of Occupational Psychology, University of Applied Sciences Bonn-Rhein Sieg, Rheinbach, Germany; 5 Department of Electrical Engineering and Computer Science, Technische Hochschule Luebeck, Luebeck, Germany

**Keywords:** Recovery, Occupational stress, Work-related rumination, Mental detachment, Smartphone-application (app), Vacation, Holidaily, App-based intervention

## Abstract

**Background:**

While work-related rumination increases the risk of acute stressors developing into chronic load reactions and adverse health, mental detachment has been suggested as a way to interrupt this chain. Despite the importance of mentally detaching from work during leisure time, workers seem to struggle to disengage and, instead, experience the constant mental representation of work-related stressors, regardless of their absence. Those who struggle with work-related rumination could benefit from an easy-access intervention that fosters mental detachment by promoting recreational activities. Especially during vacations, workers appear to naturally engage in sufficient recovery activities; however, this beneficial behaviour is not sustained. The smartphone app-based intervention “Holidaily” promotes recovery behaviour and, thus, mental detachment from work with the intension of extending the beneficial effects of workers’ vacations into their daily working life.

**Methods:**

This randomised-controlled trial (RCT) evaluates the efficacy of “Holidaily”. The Holidaily app is a German stand-alone program for mobile devices with either Android/iOS operating systems. The sample includes workers, who are awaiting to go on vacation and are randomly assigned to either the intervention (IG) or a waitlist-control group (CG). The IG receives two weeks pre-vacation access to Holidaily, while the CG receives access two weeks post-vacation. On a daily basis participants in the IG are provided with three options promoting recreational activities and beneficial recovery experiences. Online questionnaires are distributed to all participants at several timepoints. The primary outcome measure assesses participants’ work-related rumination (Irritation Scale). A significant difference two weeks post-vacation is expected, favouring the IG. Secondary outcomes include symptoms of depression, insomnia severity, emotional exhaustion, thinking about work, recovery experiences, vacation specifics, work and personal characteristics. To help explain the intervention’s effect, explorative analyses will investigate the mediation properties of the frequency of engaging in recreational activities and the moderation properties of Holidaily users’ experiences.

**Discussion:**

If successful, workers will maintain their recovery behaviour beyond their vacation into daily working life. Findings could, therefore, provide evidence for low-intensity interventions that could be very valuable from a public-health perspective. App-based interventions have greater reach; hence, more workers might access preventative tools to protect themselves from developing adverse health effects linked to work-related rumination. Further studies will still be needed to investigate whether the vacation phenomenon of “lots of fun quickly gone” can be defied and long-term benefits attained.

**Trial registration:**

German Clinical Trials Registration DRKS00013650. Registered retrospectively 15.01.2018.

## Background

### Occupational stress and health

A large body of research has established that occupational stress can negatively impact workers’ physical and psychological health. Examples of adverse effects include increased heart rate and blood pressure [1], increased risk of coronary heart disease [2] and its recurrence [3–6], as well as metabolic syndrome [7], gastrointestinal issues [8] and, especially, mental health problems like sleep complaints [9] and depression [10, 11].

### Recovery

While chronic exposure to occupational stress can lead to adverse health effects, regular periods of respite can protect workers’ wellbeing and health [12, 13]. Results of a recent meta-analysis also suggest that recovery positively influences worker performance [13]. Generally speaking, recovery can be defined as a process of unwinding, of reducing or eliminating strain caused by work stressors, thereby returning to pre-stress levels [14]. Renowned models, like the effort-recovery model [15] and allostatic load model [16], explain how the initial adaptive stress response to acute stressors may develop into chronic load and sequential maladaptive stress reactions, unless recovery takes place. Although insufficient recovery may lead to health impairment, the transition from an acute to chronic load reaction can be averted when workers recover sufficiently and replenish both their mental and physical resources [15, 17, 18].

### Work-related rumination

In turn, a risk factor that fosters the transition from acute stress to chronic load is repetitive negative thinking about work. This can be explained by the constant mental representation of work-related stressors, despite their absence [19], which is known as repetitive negative thinking. It has been assumed that repetitive thoughts prolong stress-related physiological activation [20, 21] and are a transdiagnostic risk-factor for the development and maintenance of stress-related conditions, such as depression, anxiety, sleep complaints, cardiovascular disease [22, 23], and eating and substance-related disorders [24–26]. The repetitive nature of this process is perceived as difficult to control and focuses on negative content [22] by ruminating about the past or worrying about the future [27]. When repetitive negative thinking focuses on work, it can be referred to as work-related rumination. Work-related rumination has been described as perseverative cognition, whereby repetitive thoughts focus on work-related problems [28]. Similarly, Mohr, Müller, Rigotti, Aycan and Tschan [29] label rumination as difficulty disconnecting from work during leisure time, either at home or during vacations, and named this cognitive irritation. Work-related rumination has been found to mediate the relationship between general work stress and sleep complaints [30], and the relationship between the appraisal of stressful interruptions at work and psychosomatic symptoms [31]. The constant representation of negative thoughts can be considered a severe form of insufficient detachment from work [32].

### Mental detachment

While work-related rumination increases the risk of acute stressors developing into chronic load reactions and adverse health, mental detachment has been found to buffer this relationship. Mentally disengaging and experiencing a “sense of being away” ([33], p. 579) from work during leisure time describes the process of mental detachment [34]. Although several recovery experiences are important, mental detachment has received the most research attention and been found to be particularly vital for the recovery process [12]. Among others, the Stressor-Detachment Model [35] and DRAMMA Model [36] concordantly emphasise the central role of mental detachment in protecting workers from chronic load reactions. Results from several meta-analysis indicate that mental detachment is strongly related to health indicators like depression, burnout, sleep complaints, ﻿and general wellbeing [18]. They also indicate a positive relationship between mental detachment and state of recovery amongst workers [12]. Moreover, the influence of mental detachment does not seem limited to worker health and recovery. It also can affect workers’ creativity [37, 38]. Similar associations between both work-related rumination and the lack of mental detachment and health outcomes might be explained by findings that suggest a high degree of overlap between work-related rumination and (lower levels of) mental detachment [39]. Despite the importance of mental detachment, workers who experience severe forms of occupational stress struggle, especially to mentally disengage or “switch off” from work, despite their increased need to recover [40].

### Recovery during leisure time

Physical distance from work may, in turn, foster a “sense of being away”. Studies have indicated that work breaks [41–43] and leisure time in the evenings and on weekends [44, 45] can provide promising opportunities for recovery. Moreover, engaging in recovery behaviour (i.e., recreational activities like going for walks or seeing friends/family) may reduce work-related rumination by promoting mental disengagement from work [43]. Vacations typically offer longer periods of respite [46, 47], and evidence suggests that spending time on vacation can positively effect workers’ mental health [48–50]. This is commonly termed the beneficial “vacation-effect”.

### The beneficial “vacation-effect”

With regard to change in workers’ health indicators during their vacation, in one longitudinal field study, from pre- to mid-vacation, considerable improvement was identified in scores ﻿for fatigue, satisfaction, mood, tension and energy level (Cohen’s *d =* 0.73) [46]. From a methodological point of view, this study contributes by raising awareness about the importance of assessing baseline scores 2 weeks prior to vacations, rather than immediately before. Baseline scores assessed directly before vacations can be biased, due to workers’ anticipation of their vacation [51, 52] or pre-vacation work stress [53–55]. Considering workers’ change in health indicators from pre- to post-vacation, one meta-analysis also detected an increase in positive and a decline in negative mood, exhaustion, and other negative outcomes, with a mean effect size, Cohen’s *d* = 0.43 [47]. In de Bloom et al.’s (2016) observational study, the difference in recovery between workers staying at home and those traveling was investigated and no significant difference was found between the two groups. This is similar to the results of a recently-published randomised control trial (RCT) among middle-managers, which compared short-term vacations between the intervention group, who spent their vacation away, and the control group, who remained at home [56]. Their findings indicate that 4 days away from work is sufficient for workers to experience beneficial effects in their wellbeing, strain, and recovery levels. Overall, irrespective of length and location, spending time on vacation appears to have an initial positive effect on workers’ mental health. However, this effect is not sustained after they return to daily working life.

### The short-lived “vacation-effect”

Typically within 2 weeks of having resumed work, mental health indicators return to pre-vacation levels [46, 47, 56]. Interestingly, Kühnel and Sonnentag [57] found that ﻿relaxation experiences after vacations could delay the fading of beneficial effects. This creates the impression that promoting recreational activities, which in turn foster relaxation experiences, may help sustain the beneficial vacation effect. In another recently-published, pre-post-design, intervention study, which investigated whether a mobile application could extend the beneficial effects of a vacation, meaningful improvements in mental health outcomes were noted between pre-vacation and 14-days post-vacation, where workers scores did not return to pre-vacation levels, indicating that the beneficial effect could be prolonged [58]. Consequently, while digital interventions may therefore be a promising tool, as they can be widely accessed by workers, RCT’s are needed to generate stronger evidence and provide insights into how worker recovery behaviours can be maintained beyond their vacation.

### Internet- and mobile-based interventions

To date and to the best of our knowledge, only a few RCT’s have been conducted to assess the potential of an intervention to promote recovery behaviour and reduce work-related rumination among workers. Some internet-based recovery interventions that focus on reducing repetitive negative thinking in workers have been found to have substantial effects on work-related rumination, as assessed using the Irritation Scale [29], when comparing intervention and waitlist-control groups immediately post-intervention up to 6 months follow-up [59–61]. Results indicate that the intervention’s effect on the primary outcome, insomnia severity, was found to be mediated by both work-related rumination [59] and increased recreational activities [60]. While these studies indicate the potential of digital interventions to reduce work-related rumination to a substantial and meaningful degree, they were not conducted in a vacation setting. Furthermore, internet-based interventions usually require a computer and might, therefore, be impractical for workers to access during their vacations.

#### App support for recovery

Instead, an increasingly popular way to provide behavioural interventions [62] supporting change and the maintenance of specific behaviours — like weight loss and physical activity [63], lifestyle modification among Type 1 and 2 diabetics [64] and mindfulness [65–67] — entails the use of mobile applications (apps). Apps constitute a low-threshold, scalable tool that is assumed to support behavioural change in real-world, real-time settings [68] and is promising for the delivery of mental and physical health interventions. For instance, in one meta-analysis on RCT’s, which focused on the efficacy of smartphone-based mental-health interventions for a clinical and non-clinical sample with depressive symptoms, a moderate positive effect was detected on depression relative to controls [69]. In another recently published systematic review and meta-analysis of RCT’s investigating the efficacy of standalone smartphone apps for adults with heightened symptom severity (e.g., depression, anxiety, substance use, sleep complaints), a significant effect on depression was identified relative to controls [70]. One crucial aspect of app-based interventions appears to be a good design, leading to a positive user experience. A positive user experience may determine participants engagement [71, 72], where in turn, participants engagement may determine interventions efficacy [73, 74]. The inclusion of gamification features additionally appears to enhance user engagement [75]. Recent findings suggest that users’ recovery is uniquely predicted by the user experience of a gamified app [58].

Furthermore, from a public health perspective, an app-based intervention seems promising as a tool that can be accessed easily at times and places that are most convenient for its users. On the other hand, although apps may be convenient and practical, no studies to date have investigated their efficacy promoting recovery behaviours beyond workers’ vacations.

To summarize, for the protection of workers’ mental health, it is crucial to prevent acute stress from escalating into a chronic load reaction. One major risk factor for the development of chronic load reaction is work-related rumination, since the “sense of being away” is lacking and the individual is mentally preoccupied with work-related problems. Work breaks, and especially vacations, offer opportunities for recovery, as physical distance fosters mental disengagement. Moreover, workers engage in a variety of recreational activities that promote recovery and better mental health. This effect is short-lived, however. Nevertheless, vacations may offer an ideal intervention setting, particularly since workers already intuitively engage in recovery behaviours. Encouraging workers to maintain their engagement in recreational activities beyond their vacation and to implement healthier habits when they resume work, therefore, seems the next logical step. There is evidence that internet-based interventions may reduce work-related rumination, and that mobile applications promote health behaviour changes and increase mental health. However, to the best of our knowledge, the potential of mobile applications to prolong the beneficial vacation-effect by helping workers to maintain their recovery behaviours and, thereby, experience reduced work-related rumination, has not yet been investigated.

### Research objectives

#### Primary research objective and hypothesis

The current protocol describes a randomised control trial (RCT) investigating the efficacy of the mobile application (app) “Holidaily 2.0”. It is assumed that Holidaily promotes recovery behaviour before, during and especially after vacations to prolong the beneficial vacation effect. Following the PICO framework, we hypothesise that (P-participants) workers returning to work after vacation using (I-intervention) the recovery promotion mobile application Holidaily 2.0. compared to (C-control) a wait-list control group experience (O-outcome) lower levels of work-related rumination 2 weeks after their vacation.

#### Explorative hypotheses

In addition, two explorative analyses will be conducted to investigate a potential underlying mechanism and further possible modifier of the assumed effect.

More specifically, given that levels of recreational activities are of major importance for the recovery process, they may account for the intervention’s underlying mechanism. For instance, in one intervention study, recreational activities appeared to mediate the intervention’s effect, with workers who engaged in recreational activities during lunch breaks experiencing higher levels of wellbeing at the end of their work day [43]. These findings are in line with those of an earlier RCT, in which the intervention’s effect also was mediated by increased recreational activities, leading to fewer sleep complaints [60]. In accordance with these findings and Holidaily’s intention to foster recreational activities, activity levels may explain, to a certain degree, the mechanism through which the intervention works, if it works at all.

Moreover, participants user experience might determine participants engagement [71, 72], where in turn, participants engagement may determine interventions efficacy [73, 74]. Recent findings by this study team suggest that users’ recovery is uniquely predicted by user experience [58]. On an explorative basis, we therefore intend to investigate whether users’ experience moderates the intervention’s effect.

#### Analysis of secondary outcomes and other variables

Secondary health-related outcomes will also be compared in the intervention and waitlist-control groups. It is important to note that all analyses related to secondary outcomes will be descriptive and non-explanatory in nature. As intervention research on promoting recovery after vacations is in its infancy, evaluating a wide range of secondary outcomes will help us gain knowledge to generate further hypotheses and guide future studies. Finally, all variables not directly related to health, like personal and work characteristics, are considered other variables. Other variables will be used to evaluate a potentially underlying mechanism, effect modifier and confounders of the primary study outcome. Moreover, app-use compliance will be tracked and analysed on a descriptive basis (i.e., how often was the app used).

## Methods

### Study design

For a proof of concept study and employing a pre-post-design, our research team has investigated the feasibility of use of the app-based intervention “Holidaily 1.0.” amongst workers (*N* = 77) [58]. Holidaily is a low-intensity, gamified, smartphone-based intervention. The app provides users with three suggestions promoting recovery behaviour daily. In this study, the intervention was begun 2 weeks prior to each worker’s vacation and continued beyond their return to daily working life. Initial findings were promising, as workers reported a significant improvement from 2 weeks before to 2 weeks after their vacation, and exhibited reduced work-related rumination during their leisure time, with an effect size of *d* = 0.68. To date, research indicates that, with no intervention, mental health variables resume baseline scores within 2 weeks after a vacation has ended [47]. Our next step is to establish whether meaningful effects can also be detected when an intervention and control group are compared.

Therefore, the current two-armed randomised controlled trial is being conducted to evaluate the efficacy of the app-based intervention “Holidaily 2.0” relative to a waitlist-control group (CG). All participants are invited via email to complete online questionnaires 2 weeks prior to their vacation, on their last working day, in the middle of their vacation, at the end of their first day working post-vacation, and 2 weeks after their vacation has ended (see Table 1). The intervention group receives an additional questionnaire 4 weeks after their vacation (extended follow-up). On average, these assessments last 15–30 min each. After the CG complete their final questionnaire, 2 weeks after their vacation, they receive an automated code via Email which allows them to immediately access Holidaily.

**Table 1:** Overview of variables and measuring time points

	**Measuring time points**					
	**Two weeks pre-vacation (T1)**	**Last working day (T2)**	**Mid of vacation (T3)**	**First working day (T4)**	**Two weeks post-vacation (T5)**	**Four weeks post-vacation (T6**^**a**^**)**
**Measures**						
*Demographics*	✔					
*Vacation specifics*	✔	✔		✔		
**Primary outcome**						
Work-related rumination						
*Work-related rumination (IS)*	✔	✔	✔	✔	✔	✔
*- cognitive irritation*						
**Secondary outcomes**						
Mental health						
*Symptoms of depression (PHQ-8)*	✔			✔	✔	✔
*Insomnia severity (ISI)*	✔	✔	✔	✔	✔	
Work related health						
*Emotional exhaustion (MBI)*	✔			✔	✔	✔
*Work-related rumination questionnaire (WRRQ)*						
*- affective rumination*	✔	✔	✔	✔	✔	
*- problem-solving pondering*	✔	✔	✔	✔	✔	
*Recovery experiences (DRAMMA-Q)*						
*- mental detachment*	✔	✔	✔	✔	✔	✔
*- relaxation*	✔	✔	✔	✔	✔	✔
*- autonomy*	✔	✔	✔	✔	✔	✔
*- mastery*	✔	✔	✔	✔	✔	✔
*- meaning*	✔	✔	✔	✔	✔	✔
*- affiliation*	✔	✔	✔	✔	✔	✔
**Other variables**						
Work characteristics						
*Unfinished tasks*	✔	✔		✔	✔	
*Time pressure (ISTA)*	✔	✔		✔	✔	
*Work availability during vacation*	✔			✔	✔	
*Working overtime*	✔					
*Task variety (WDQ)*	✔					
*Social support at work (SzSU)*	✔					
Personal characteristics						
*Work engagement (UWES)*	✔	✔		✔	✔	✔
*Work performance (OCB)*	✔	✔		✔	✔	
*Boundary management (WLI)*	✔					
*Work-Life-Balance (TKS-WLB)*	✔	✔			✔	✔
*Life-satisfaction (SWLS)*	✔					
*Resilience (RS)*	✔				✔	
*Vitality (PANAS)*	✔	✔	✔	✔	✔	✔
*Physical health*	✔					
*Wellbeing*	✔	✔	✔	✔	✔	
*Need for recovery (NFR)*	✔	✔		✔		✔
*Creativity (objective) (TTCT)*	✔	✔		✔		
*Creativity (subjective)*	✔	✔		✔	✔	
**Potential mediator and moderator**						
Mediator						
Health behaviour						
*Recreational activities (REAQ)*	✔				✔	✔
Moderator						
Technical aspects						
*User experience (AttrakDiff2)*					✔	✔

^a^ only intervention group

All procedures were approved by the ethics committee of Leuphana University in Lueneburg (reference number: 201606, EB-Antrag Lehr201606_holidaily). The trial is registered with the German Clinical Trial Register (DRKS00013650).

#### Inclusion and exclusion criteria

To participate in the study, prospective applicants must sign up at least 14 days prior to their vacation. They also must be gainfully employed, at least 18 years of age, a smartphone user with internet access, willing to give informed consent, returning to work after their vacation, and indicating, with a score of ≥14 on the Irritation Scale, higher levels of work-related rumination [76]. This last criterion was chosen to compare our results against those of previous studies that employed this criterion [60, 61] and only address those workers who struggle with mentally disengaging from work [40]. A further requirement is that all participants must be able to read and write German to complete the questionnaires. Participants are excluded if they are taking part in other recovery/stress training, engaging in psychotherapy, or using variable-dose medication for sleeping complaints.

#### Procedures

Recruitment and study execution have been taking place since the summer of 2017 and will continue until roughly the winter of 2021. Participants have been and will continue to be recruited via newspaper announcements, on-air media and related websites. Individuals interested in participating in the study can register anonymously online at www.holidaily.de by providing the research team with their e-mail address or by sending an e-mail to the research team directly. Potential participants receive an email with study information.

**Fig. 1:**
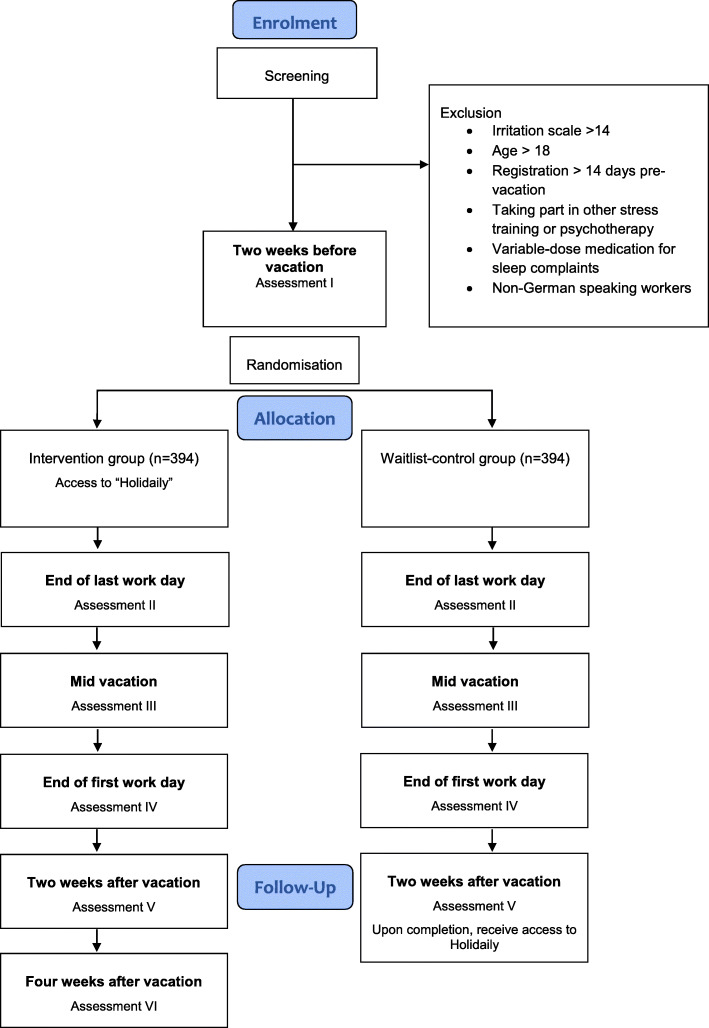
Study flow chart

Prospective participants must complete an online screening questionnaire that includes questions to assess their level of work-related rumination and vacation period, and provide informed consent. The informed consent form provides details on the background, objectives and procedures of the study. Participants are assured that all data will remain confidential and that they have the right to withdraw from the study at any time. Participant identifiers will only be available to those conducting the study. Anonymised information will be used for analysis. Subjects are included in the study if they fulfil all the inclusion criteria and none of the exclusion criteria. They are then randomly allocated to one of two study conditions (see Fig. 1). Randomisation takes place once participants have completed the baseline questionnaire. Participants who fulfil the criteria will be randomly allocated in blocks with variable block length at a 1:1 ratio to the intervention or the control group. This balanced randomisation technique should ensure an equal distribution of participants into both groups. This concealed allocation sequence takes place automatically through the online platform Unipark (Questback, Cologne, Germany).

#### Assessment and allocation to the intervention

Participants are randomised to the conditions through a computer-generated algorithm created by Unipark. The allocation sequence is concealed to the researchers until interventions are assigned. For each measurement timepoint, participants receive an individual link via email that invites them to complete the online questionnaires at Unipark. Participants in the intervention group receive a personalized reminder 2 weeks after their vacation via email, asking them to complete the final questionnaire. All study-related information will be stored securely at the study site and handled with great care. Data are recorded and evaluated pseudonymised and transmitted in encrypted form. Data are stored on the server of Leuphana University in Lueneburg. Neither personal photos nor the content of notes in the app is transmitted or analysed. Only the amount of Dailys will be recorded for each participant.

#### Sample size calculation

Sample size calculation was based on the expected standardized difference in the primary outcome measure — work-related rumination (Irritation Scale, [76]) — between the intervention and control groups 2 weeks post vacation. Evidence from meta-analyses and systematic reviews on the efficacy of digital interventions for mental health, and specifically stress management, in workers indicate an effect size of ﻿*g* = 0.54 [77]. More precisely, data on mental health apps as a standalone treatment for clinical samples targeting various conditions yield mixed results: for instance, a significant effect for depression ﻿*g* = 0.33, but no effect for ﻿anxiety [70]. Few RCTs indicate the potential of any app-based intervention targeting stress-related complaints amongst workers [65, 66, 74]. Though clear results concerning the efficacy of app-based interventions cannot be drawn, findings nonetheless indicate their potential. From a public health perspective, the aim is to reach the whole population. As such, smaller effects are meaningful, as more people can potentially benefit from an intervention. Taking these two lines of reasoning together, we regard an effect of *d =* 0.20 for work-related rumination as meaningful, especially when considering that the app constitutes a low-intensity self-help intervention for the general working population. A priori G*Power analysis [78] for a two-tailed test, in which 80% power and a 5% significance level are assumed, indicates that an overall sample size of *N* = 788 is needed to detect differences statistically.

### Intervention content and app design

The Holidaily app is a stand-alone program for mobile devices with either Android or iOS operating systems. Holidaily is available in German, Finish and English and was developed along several lines of research. Its key components are prompts for recreational activities, called “Dailys”. Dailys are based on behavioural activation strategies and refer to specific activities that positively influence mental health [79]. To promote behavioural change, appropriate behaviour-change techniques — like health-development tracking and prompts — were included [80]. An important element of the gamified app is its potential to reward participants by enabling the collection of points in exchange for completing Dailys and tracking one’s wellbeing (for examples, see Additional file 1).

#### Holidaily’s key features

The Holidaily-app consists of five sections (for examples, see Additional file 2). First, on the “***Home***” screen, users can keep track of their collected points and navigate directly to “Dailys”. The more points the user collects, the “richer” the background of the home screen appears. The avatar on the home screen also reflects whether users are on vacation or not. This is indicated by the avatar either holding a cup of coffee and wearing a business-like outfit, representing daily working life, or holding a cocktail and wearing a flower chain and hula skirt to indicate that they are on vacation.

The second section consists of “***Dailys***”. This is the main section, with each Daily corresponding to at least one of the six mechanisms of the DRAMMA model [36]. The various exercises differ before, during, and after vacation. Dailys foster each mechanism to a different degree of intensity, duration and effort. This section consists of three features: (i) Users choose and “book” one Daily from three suggestions or, alternatively, generate their own recovery activity. Users can also place Dailys “on hold” to be carried out at a more convenient time. (ii) After the user completes the respective exercise, they are asked to rate it, based upon the six DRAMMA recovery experiences. Users rate the extent to which the Daily helped to foster the experiences of detachment, relaxation, autonomy, mastery, meaning and affiliation. The mean score of the user’s rating is displayed graphically in the third section, called “***Recovery***”. Encouraging users to rate their recovery experience fosters reflection over which particular activity was helpful for the individual’s recovery experience, while also encouraging perceived recovery self-efficacy. As a reward, users receive points for each completed Daily; (iii) users are presented with an overview of all “on hold”, “booked”, and “done” Dailys. The fourth section, “***Feel***”, invites users to rate their wellbeing using three validated, one-item questions regarding their experience of their past day, mood and energy level [81–83]. Users are encouraged to complete this section on a daily basis. The fifth section, “***Recovery***”, provides users with a summary of their recovery profile and has three sub-sections. Graphs visualize users’ recovery and wellbeing history, as well as their individual recovery strengths. This information is based on data entered by users. The section aims to promote users’ self-reflection, self-monitoring, and perceived self-efficacy.

#### Additional features

Holidaily offers additional features, like information about the vacation location, weather forecasts, a walkthrough, and a section with additional information about the app’s scientific background. Pictures can be uploaded to individualize Holidaily’s interface and keep track of the completed Dailys (i.e., users can upload a photo and write a short note about how they completed the Daily). The Daily overview can serve as a diary and users can always go back to view their completed Dailys.

#### Improving adherence to the intervention

Several steps have been taken to improve participants’ adherence to intervention protocols and reduce non-responses, as suggested by Newman [84] (Additional file 2). For example, Holidaily sends push-messages on a daily basis to remind users to complete Dailys and rate their wellbeing. To further foster engagement in recovery behaviour, participants can search for specific Dailys that were particularly helpful at promoting experiences of recovery via a sorting function within the app. Users also receive a weekly feedback email that summarises their recovery development.

#### User experience

As high user exposure rates are considered pivotal to an app’s effectiveness [85], aspects highlighted by the Mobile App Rating Scale (MARS: [86]) were considered when designing Holidaily. Special focus was given to attain good user experiences, as they are considered by MARS to be highly influential, in terms of determining the quality of the app promoting behavioural change [86]. More specifically, recent findings by this study team support this claim and that a user’s recovery seems to be uniquely predicted by the user’s experience with the app [58]. Furthermore, an app review that investigated the use of gamification techniques in stress management apps identified 17 commonly-used gamification features [87]. Of these 17 features, nine were considered during the designing of Holidaily (see Additional file 1). These, for instance, include the avatars called “Holidaisy” and “Holidave”, which reflect users’ wellbeing.

### Intervention

#### Holidaily-app intervention group

Participants in the Holidaily-app intervention group (IG) receive access to the Holidaily-training app 3 weeks before their vacation is due to start. After logging in, users create an individual profile for their upcoming vacation. This step includes entering the following data: vacation location, first and last day of work, and first and last day of vacation. Finally, users answer three questions related to each of the six DRAMMA dimensions. These questions will make it possible to compare their actual and desired levels of recovery experiences 2 weeks after returning from their vacation. After they have generated a personal vacation profile, users can start using the app, completing and keeping track of their Dailys and wellbeing.

#### Waitlist-control group

Participants in the waitlist-control group (CG) are invited to answer the same questionnaires following the same timeframe as the IG. However, subjects in the CG only receives access to Holidaily 2 weeks after they have returned to work. Controls can, therefore, use Holidaily during their subsequent daily working life and next vacation.

### Outcome measures & potential mediator and moderator

Work-related rumination after vacation is considered the primary outcome measure. Secondary outcome measures include mental health and work related health, and are only descriptive and non-explanatory in nature. Further variables not directly related to health, like personal and work characteristics, are defined as other variables. Table 1 provides an overview of all instruments applied at each measurement time point. Participants are asked to respond to all questions thinking about the previous week, unless otherwise clearly stated. All self-report measures are assessed in German.

#### Demographics

A self-designed questionnaire is used to collect general and vacation specific demographic data 2 weeks prior to the vacation; for instance, participant’s age, sex, relationship status, number of children, educational background, work status (part/fulltime), work hours per week per their contract, and actual working hours, length of employment, whether or not they have a managerial position, frequency working from home, last/first work day, and first/last vacation day.

#### Vacation specifics

Directly before participants leave for their vacation, they are asked to indicate their anticipated work accumulation during their absence (on a scale 1–5), type of vacation, who they are going on vacation with and how good their relationship is (on a scale 1–10), do they perceive they have control regarding the organisation of their vacation (on a scale 1–10), what is their vacation destination and have they been there before, and what are their expectations regarding their vacation. Further vacation specific questions are asked directly after their vacation, including did they enjoy their vacation (on a scale 1–10); what was the weather like; did they have negative/positive experiences during their vacation (open-ended question); how many hours did they work during their vacation; and, if so, did they enjoy it and experience control over their work (on a scale 1–5); what did their work involve; and to what extent and how often were participants contacted by colleagues, clients or superiors during their vacation.

### Primary outcome measure

#### Work-related rumination

Work-related rumination is measured using the cognitive irritation subscale of the Irritation Scale (IS) [76]. This subscale consists of three items, each with a 7-point Likert response scale (range 0–6) to assess workers’ ability to mentally detach from work during leisure time. The level of internal consistency previously reported was Cronbach’s *α* = 0.89 [76].

### Secondary outcome measures

All secondary outcomes are health-related.

### Mental health

#### Depression

Symptoms of depression is assessed with the Patient Health Questionnaire (PHQ-8) [88]. This instrument consists of eight items. Participants indicate how “down, depressed, or hopeless” they feel on a 4-point Likert response scale (range 0–3). Internal consistency has been reported as *α* = 0.84 [89].

#### Insomnia severity

Insomnia severity is assessed using four items from the Insomnia Severity Index (ISI) [90]. For example, participants are asked to rate, on a 5-point Likert scale (range 0–4), how much they struggled to sleep during the night. A summation score from participant’s answers is calculated. ﻿Internal consistency of this measure has been found to be *α* = 0.74 [90].

### Work-related health

#### Emotional exhaustion

Emotional exhaustion is being measured with the Maslach Burnout Inventory General Survey (MBI-GS-D). Five items from the exhaustion subscale, assessed on a 6-point Likert scale (range 0–6), are used. Participants rate statements indicating whether their work made them feel emotionally drained or exhausted. The internal consistency of this measure has been reported as *α* = 0.85 [91].

#### Thinking about work

Further aspects related to thinking about work are assessed using the work-related rumination questionnaire (WRRQ) [92]. The subscale, affective rumination, consists of five items and assesses workers’ negative and reoccurring thoughts about work; for example, “﻿do I feel irritated by work issues when not at work?” The second subscale, problem-solving pondering, also consists of five items and assesses workers constructive thoughts about how they can improve their performance; for example, “after work, I tend to think of how I can improve my work-related performance”. All items are rated on a 7-point Likert response scale (range 0–6). Internal consistency has been found to be *α* = 0.90 for affective rumination and *α* = 0.81 for problem-solving pondering [93].

#### Recovery experiences

Participants’ recovery experiences are measured employing the DRAMMA questionnaire (DRAMMA-Q), which consists of six subscales measuring detachment, relaxation, autonomy, mastery, meaning and affiliation. All 18 items are rated on a 5-point Likert scale (range 0–4). DRAMMA-Q was based on existing, validated questionnaires, and has itself been validated [94].

### Other variables

The following measures are not directly related to mental health.

### Work characteristics

Various work characteristics of participants are also assessed. Unless otherwise stated, questions refer to the past 7 days. Work engagement is assessed using the Utrecht Work Engagement Scale (on a scale 1–7) (UWES: [95]). Other aspects such as, vitality (PANAS: [96, 97]) (on a scale 1–7), perceived need of recovery (NFR: [98]) (on a scale 1–5), and objective (TTCT) and subjective creativity (on scales 1–5) [99–101] are also assessed. Further measures include the perceived number of unfinished tasks (on a scale 1–5) [102]; work performance (e.g., did participants accomplish tasks that were beyond their job description?) (on a scale 1–7) (OCB: [103]); and whether participants experienced time pressure (on a scale 1–5) (ISTA: [104]. We also take into consideration whether participants experience autonomy in the order in which they completed their tasks, as well as the variety of tasks at hand (on a scale 1–5) (WDQ: [105]), and whether they perceive receiving social support from their colleagues (on a scale 1–5) (SzSU: [106]).

### Personal characteristics

For assessing workers ability to manage boundaries between their work and private life, we employ the Work-life Indicator (WLI) [107] and Work-Life-Balance scale (on a scale 1–6) (TKS-WLB: [108]) (on a scale 1–6), as well as the Life Satisfaction Scale (on a scale 1–7) (SWLS: [109]). Participants perceived levels of physical health and subjective wellbeing are measured (see for instance, [46]). Personal characteristics include resilience (RS) (on a scale 1–5) [110].

### Potential mediator, moderator

#### Mediator

##### Health behaviour: Recreational activities.

The frequency of participants’ engagement in recreational activities after work during the past week is assessed using 21 items from the Recreation Experience and Activity Questionnaire (ReaQ) [111]. Items are rated on a 5-point Likert scale (range 0–4) and have an internal consistency of ﻿*α* = 0.88 [61].

#### Moderator

##### Technical aspects: User experience.

Participants’ digital experiences using Holidaily are assessed employing a 28-item questionnaire from the AttrakDiff2 [112]. A seven-point Likert scale is used for responses. The questionnaire provides information concerning global attractiveness (ATT), pragmatic quality (PQ), hedonic quality-identity (HQ-I), and hedonic quality-stimulation (HQ-S), and has an internal consistency of ﻿*α* = 0.94 [58].

### Statistical analyses

﻿All analyses will be reported according to the Consolidated Standards of Reporting Trials (CONSORT) for web-based and mobile health interventions [113], employing intention-to-treat (ITT) procedures*.* All reported *p* values will be two-tailed, with α = 0.05 the chosen significance level. Characteristics of the sample will be analysed using descriptive statistics. For the primary and all continuous secondary outcomes, Cohen’s *d* will be calculated based on group differences in means post-vacation, standardized by the pooled standard deviation of the post-vacation scores. Missing data will be dealt with following the recommendations of Little and Rubin [114] and Schafer [115]. Multiple imputation (MI) is an especially robust approach for handling missing data [116]. A stability score, in form of correlation analyses, will also be reported in the results section.

### Primary analysis

The primary analysis will evaluate the efficacy of Holidaily. The primary endpoint is the difference in work-related rumination, assessed using the irritation score, between the IG and CG 2 weeks after the vacation. We will use analysis of covariance (ANCOVA) to investigate differences in the primary outcome between the two groups, as simulation studies have demonstrated that ANCOVA is the most robust method for analysing RCT’s, in terms of protecting against bias, while enhancing precision and statistical power [117, 118]. Baseline scores for the primary outcome will be included as a covariate. In addition, for the analyses of differences in work-related rumination at the end of the last work day, mid-vacation and at the end of the first work day Bonferroni-Holm corrections will be used to reduce the chances of chance capitalisation [119].

### Sensitivity analyses

To assess the robustness of these results, sensitivity analyses will be conducted. First, a separate analysis will be performed for participants who complete the main assessment 2 weeks after vacation (study completer sample). Second, all participants that used the app at least three times per week will be analysed (per protocol sample). Third, to account for potential baseline differences, despite randomisation, we will include all continuous variables with a between group difference of *d* = 0.20 or more and each categorical variable with 10% difference or more as covariates.

### Secondary analyses

Secondary analyses will include further ANCOVAs for each mental health and work related health variable. Baseline scores for the respective independent outcome will be included as a covariate to control for potential baseline differences.

### Further analyses

#### Mediation analysis

To assess the mediating role of proposed variables for the intervention’s effects on work-related rumination, mediation analysis will be performed [120]. As Holidaily encourages users to engage in recreational activities, these may have increased 2 weeks after vacation among those in the IG. To attain temporal precedence, the change in reactional activities will be calculated (post-vacation minus pre-vacation scores). An indirect effect is considered significant if its 95% confidence interval excludes zero.

#### Moderation analysis

In addition, when employing mobile interventions, it may appear relevant to investigate whether users’ technical experience moderates the intervention’s effect. For this reason, participants’ user experience will be considered as a moderator. To detect significant moderation, the Johnson-Neyman (J-N) [120, 121] procedure will be used to pinpoint at which moderation value a meaningful difference between groups is observable in the primary outcome.

## Discussion

### Study objectives

This study protocol describes a randomised control trial (RCT) examining the efficacy of a newly-developed behaviour change smartphone app, called Holidaily. Holidaily encourages workers to engage in recreational activities before, during and especially after vacations to ideally prolong the beneficial effects of vacationing. We expect levels of the primary outcome, work-related rumination, to be significantly lower 2 weeks post vacation in the intervention versus waitlist-control group. This finding would be important, especially because the constant mental representation of stressors, despite their absence, has been found to extend stress-related physiological activation and, thereby, contribute to the development and maintenance of mental illness. In addition, the hypothesised mechanism — that a change in recreational behaviour causes the effect on work-related rumination — will be investigated via mediation analysis.

### Study contributions

Particularly because intervention research on the promotion of recovery after vacations is in its infancy, the present study can contribute to the emerging field in several ways.

First, to the best of our knowledge, this is the first RCT to investigate the efficacy of an app-based intervention encouraging workers to improve and maintain their engagement in recreational activities, with the aim of reducing work-related rumination beyond their vacation into daily working life.

Second, implementing an intervention that promotes recovery behaviours during a period when workers naturally engage in restorative activities may be promising. A systematic review of behaviour theories by Kwasnicka et al. [122] suggests that initiating behaviour change should occur when an individual’s motivation level is high and their opportunity costs are low. Especially during vacations, it appears that workers can effortlessly create opportunities and are naturally invested, as well as motivated, to engage in recreational activities [46, 47]. Although vacations seem to offer a promising starting point for implementing a behaviour change intervention, this has not yet been investigated. Our study’s findings may, therefore, contribute by assessing the value of promoting behaviour change maintenance during and beyond workers’ vacations.

Third, so far, only high-intensity interventions — such as personally-guided web-based training — have been investigated and found effective at reducing work-related rumination (i.e., [61]). Consequently, little is known about app-based interventions’ potential when targeting the reduction of work-related rumination. From a public-health perspective, app-based interventions may be a favourable alternative, as internet-based interventions usually require a laptop computer, which might be inconvenient for workers to access during their vacations. As such, app-based interventions may be more practical, since they can be accessed flexibly and independent of time and place. Our study could, therefore, contribute to this research field by providing insights into the effectiveness of app-based interventions.

Fourth, investigating a potentially underlying mechanism may extend our understanding of the efficacy of low-intensity interventions and health behaviour. Mediation analysis will assess whether change in recreational activities mediates the app’s effect on work-related rumination.

Fifth, exploring whether users’ technical experience with Holidaily contribute to a greater intervention effect could be of great value when designing future app-based interventions. For this reason, moderation analysis will be performed, focusing on Holidaily users’ experiences and the intervention’s effectiveness.

### Limitations

Despite the potential contributions of the present study, several limitations must be considered. First, this study’s recruitment strategy involves public media, including Facebook, Instagram, and both radio and TV interviews. Prior to receiving access to Holidaily, the waitlist-control group might become aware of the intervention’s primary aim: to promote the maintenance of recovery behaviours beyond vacations. This could, perhaps, lead to behavioural changes in the waitlist-control group, as well. If this happens, the intervention’s effect could be diffused [123], indicating only small differences between the two groups at 2 weeks after vacation, leading to underestimation of the app’s true potential. Although public media might reduce the study’s internal validity, external validation of the app might be greatly improved. By broadcasting about the intervention via public media, a potentially wider range of workers could learn about public health measures and may, thus, access health interventions like Holidaily. As a result, the study’s samples may become more heterogenous. Overall, recruiting via public media mimics real-life implementation of health promotion and, therefore, increases the external validity of the current study.

Second, relative to high-intensity interventions and clinical studies, the expected effect size could be considered relatively small. However, this interpretation might be misleading. Clinical studies commonly target mental illnesses like depression and anxiety [124], where a greater range of improvement is expected. Conversely, this study targets a risk factor in the general working population for which a smaller degree of improvement is anticipated. This has been described as the prevention paradox [125], stating: “however much it may offer to the community as a whole, it offers little to each participating individual” ([125], page 1850). Accordingly, health-promotion strategies might only offer a small amount of benefit per individual, but exert a major societal impact by reaching a large population. At a population level, promoting the reduction of work-related rumination may contribute to the prevention of mental illnesses and may, therefore, be beneficial to a larger population. In turn, this also could reduce the costs and time otherwise spent on later treatment for mental illnesses triggered by work-related rumination.

Third, findings from previous observational studies suggest that the beneficial vacation effect experienced by workers is not sustained, but fades out shortly after they return to work, with mental health indicators returning to pre-vacation levels [46, 47, 56]. Consistent with previous investigators, who usually selected 2 weeks to describe the fading effect, we maintained this timeframe for our primary measurement. Additionally, a 2 week extended follow-up also is included, albeit only to assess stabilisation after the vacation in the intervention group. Further investigations are, therefore, needed. While 2 weeks may be insufficient to verify lasting behavioural changes, they might provide insights into whether the fadeout effect can be reduced. If Holidaily appears to be effective in the short-term only, future studies should also consider potential facilitators and barriers for long-term maintenance.

In the present study, three possible barriers might limit the intervention’s effect to two-weeks. For instance, after their vacation, workers may experience reduced motivation and increased effort to engage in recovery behaviours, due to stresses encountered at work (e.g., work accumulation during their absence; see also [57]). This might lead to an initial relapse, prompting the fading of the beneficial effect. To overcome such barriers, certain behavioural change techniques, like prompts and self-monitoring, were integrated into this intervention to promote engagement in recovery behaviours during daily working life. It is unclear whether these are appropriate strategies, however. To assess changes and control for potential confounders, for instance, workers’ motivation/engagement is being measured by the Utrecht Work Engagement Scale [95]

Second, the length of workers’ vacation may play a greater role determining the maintenance of behavioural change than previously anticipated. In one study, as few as 18 or as many as 254 days are required before a newly adopted health behaviour — like improved diet or regular exercise — becomes stable and replaces previously dominant behaviours [126]. Contrastingly, workers in Germany are legally entitled to a minimum of 24 days of paid vacation. Although the length of workers’ vacation period may be long enough to initiate short-term behavioural change, it might be insufficient for benefits to be maintained long-term. Hence, vacation length will be considered as a confounder.

Third, contrary to studies that suggest that fading of the beneficial vacation effect could be related to barriers hindering behaviour change maintenance, in fact this could be an expression of hedonic adaptation. According to Norrish and Vella-Brodrick [127], individuals quickly adapt to change, beneficial effects and novel situations. To counteract adaptation, literature highlights the importance of variety [128]. Although varying recreational activities are integrated into the intervention, perhaps further digital elements — like audio and video features demonstrating specific recovery exercises, or the capacity to upload a video recording from their vacation — could be useful.

Finally, a strong conclusion about the efficacy of the intervention can only be drawn for the primary outcome, the effect of Holidaily on work-related rumination. Results for all secondary outcomes and other variables are descriptive in nature and should therefore be interpreted with caution. However, as interventional research aiming to prolong the beneficial effects of vacation is in its infancy the broad assessment of secondary outcomes should expand the knowledge in this under researched field and guide future research.

## Conclusions

In summary, this study uses a naturally occurring emotional highlight, workers’ vacations, as a starting point to encourage workers to maintain their beneficial health behaviours beyond their return to work. If successful, this study’s findings could provide evidence for low-intensity interventions to promote mental health amongst workers. App-based interventions have an especially great reach. Consequently, more workers could access preventative tools to help protect them from mental illness linked to work-related rumination. Finally, the strategy of using positively-tuned events (i.e., vacations) to implement health promotion might also be a viable option for addressing other health behaviours.

## Supplementary information

**Additional file 1.** Appendix 1. Behaviour Change Techniques implemented in Holidaily and Gamification feature implemented in Holidaily. Description of data: The included date shows examples of which/how behaviour change techniques and gamification features were been implemented/employed in the Holidaily app

**Additional file 2.** Appendix 2. Description of data: Date shows five example images of what users would see when using Holidaily, for instance, Holidaily’s “Home”–screen, one particular “Daily” and participants “Recovery” process in form of a diagram.

## Data Availability

The datasets used and/or analysed during the current study are available from the corresponding author upon reasonable request.

## Abbreviations

RCT: Randomised control trail
App: Smartphone application
IG: Intervention group
CG: Waitlist-control group
