# Supplementary material for: Efficacy of a smartphone-based intervention — “Holidaily” — promoting recovery behaviour in workers after a vacation: study protocol for a randomised controlled trial
Source: BMC Public Health. 2020 Aug 26;20:1286. doi: 10.1186/s12889-020-09354-5 (PMC7448331; doi:10.1186/s12889-020-09354-5)
Supplement: Supplementary file 2 — Additional file 2. Appendix 2. Description of data: Date shows five example images of what users would see when using Holidaily, for instance, Holidaily’s “Home”–screen, one particular “Daily” and participants “Recovery” process in form of a diagram. [file 12889_2020_9354_MOESM2_ESM.docx]

Appendix 2.

First: “***Home”*** *-* *the avatar reflects where the user is, i.e. on vacation or during daily working life.*


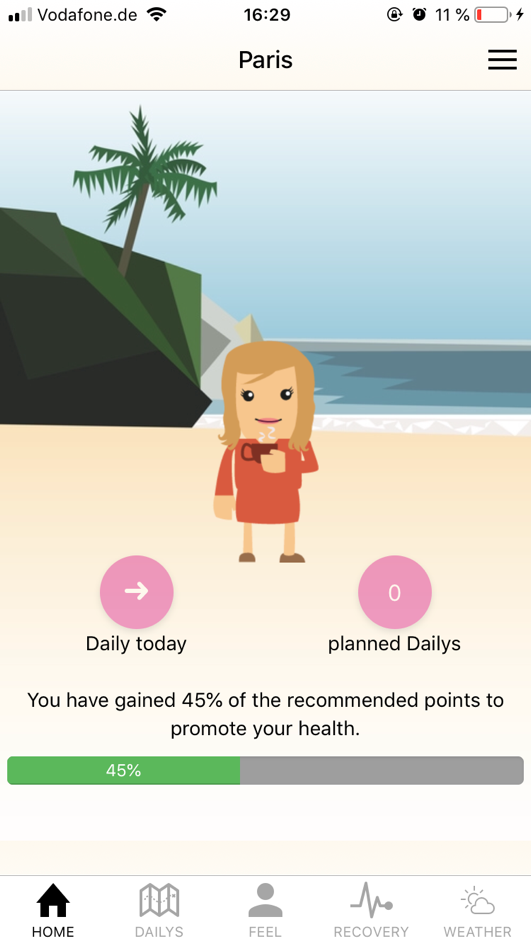


Second: ***“Daily”*** *-* *Example*


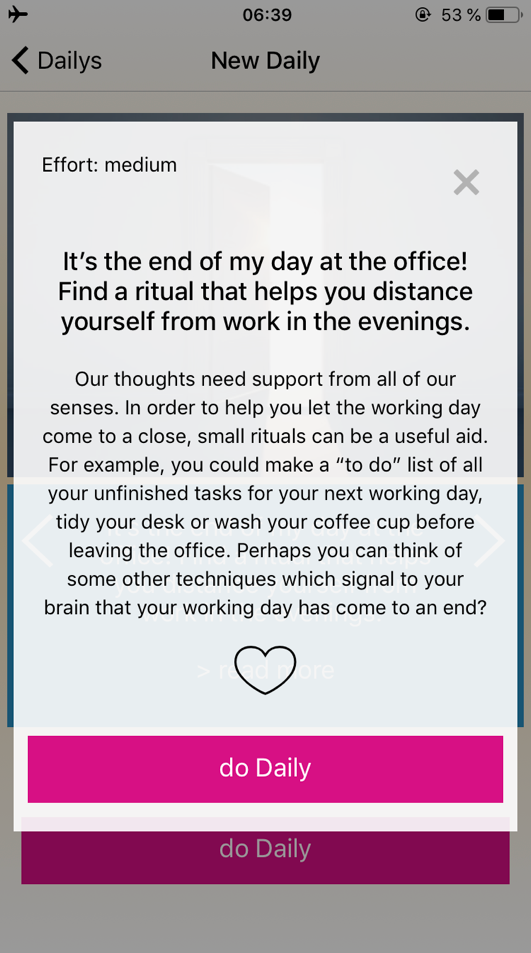


Take your mind off work!

After scheduling some time to unwind, be sure to also engage sufficiently in other activities to help take your mind off work. Perhaps seeing friends or engaging in your favourite exercise will help you.

***
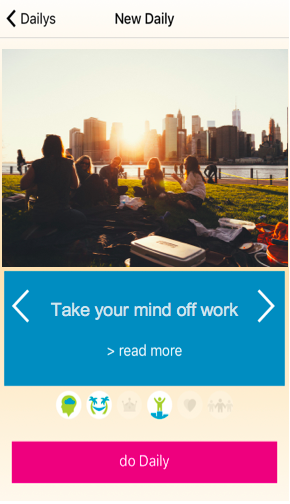
***

Third: “***Feel”*** *-* *these values are displayed in the recovery section “Wellbeing”*

***
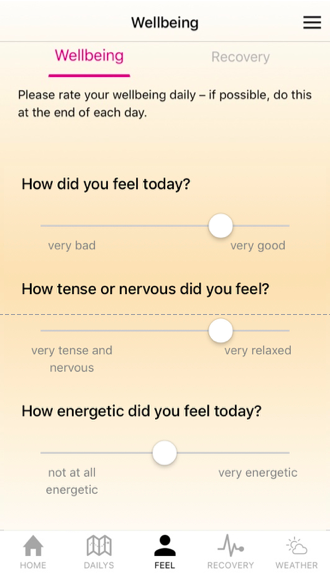
***

Fourth: ***“Recovery”*** - *after completing each Daily users rate their recovery experience along the six Dramma mechanisms. The mean score is represented in the below graph.*

Expectation of the recovery for after the vacation

Expectation of recovery for during the vacation

Flow of actual recovery

Recovery entry


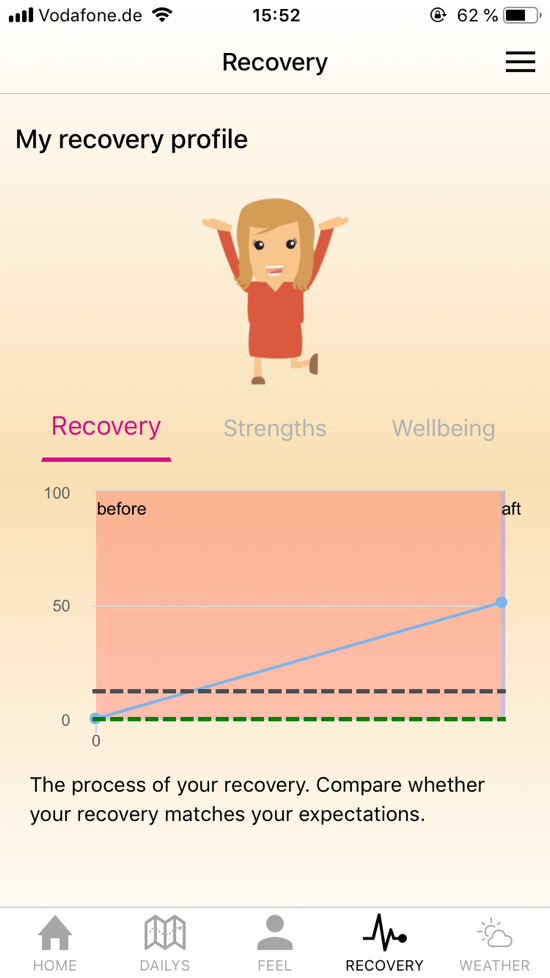


before

during

after
